# The role of medication timing and patient involvement in adherence to treatment for hypertension

**DOI:** 10.1038/s41598-025-98583-y

**Published:** 2025-10-07

**Authors:** Arzu Akbaba, Hasan Fehmi Dirik, Hatice Mert

**Affiliations:** Faculty of Nursing, Dokuz Eylul University, Izmir, Turkey

**Keywords:** Hypertension, Medication adherence, Medication timing, Nurse, Patient involvement, Cardiology, Health care

## Abstract

Despite improvements in the effectiveness of medication for the treatment of hypertension, patient adherence to prescription regimens remains a challenge in clinical practice. This study examined the impact of two factors on improving medication adherence: medication timing (morning timing is considered as 06:00–10:00, and evening timing, as 20:00-midnight) and patient involvement. Following the STROBE guidelines, a cross-sectional survey design was undertaken at a university hospital between May and August 2023. Three hundred and four inpatients with a diagnosis of hypertension comprised the study sample. Data was gathered using the Control Preferences and Medication Adherence Report Scales. The results revealed that patients preferred to take a passive role in taking their medications (60.2%). However, more active patient participation in decision-making resulted from considering patients’ daily routines (80.9%) and positive attitudes from healthcare providers during communication (69.4%). Regression analyses indicated that patient involvement (OR:5.649, 95%CI [2.528–12.622]), rather than medication timing (OR:1.052, 95%CI [0.591-1.875]), predicted treatment adherence. Shared decision-making improved adherence to medication regimes compared with passive acceptance (81.0% vs. 50.8%; *p* = .000). These findings underscore the significance of patient involvement. Given patients’ preference for a paternalistic approach, in which healthcare providers make the decisions, often without patients’ active involvement, promoting participation through strategies such as providing ample time for treatment discussions, offering information, and aligning treatment hours with patients’ daily routines are promising approaches for enhancing adherence.

## Introduction

Hypertension is a prevalent disease worldwide and is a significant risk factor for cardiovascular diseases^1^. Despite the existence of effective treatments for hypertension, most patients fail to reach their target blood pressure levels; a crucial factor is adherence to prescribed antihypertensive drugs^1–3^. According to the World Health Organization, the adherence rate of patients with hypertension to drug treatment is only 21% in developed countries^4^. Similarly, in Turkey -a developing country-, 57.3% of patients receive antihypertensive medication, but only 31.5% achieve their target blood pressure levels^5^. Patients often miss their scheduled check-ups, neglect to take their medications as prescribed^6^, and discontinue drug use within a few months of starting treatment^1^. Therefore, promoting adherence to drug therapy is critical for patients with hypertension to maintain their blood pressure within the desired range and prevent potential complications^7^.

Adherence to hypertension treatment can be influenced by a variety of factors, both positive and negative^8^. To explain the factors contributing to medication adherence, complementary to those identified by Gutierrez & Sakulbumrungsil (2021), Peh et al. (2021) proposed a conceptual model encompassing five main domains based on 102 conceptual frameworks^8,9^. Factors contributing to better adherence include good relationships between healthcare providers and patients, easy access to healthcare services, and specialized clinics and programs, while factors having an adverse impact include a low level of education, unemployment, and living alone, namely, socio-economic characteristics. Other patient-related factors include low health literacy, awareness and knowledge about hypertension, inappropriate attitudes toward hypertension, low self-efficacy, and lack of social support. Adherence can also be affected by therapy-related factors, such as inconsistent drug regimen schedules and competing complementary and alternative medicines. The final category is condition-related factors, such as disease impact on lifestyle and comorbidities^8,9^. To improve adherence, it is essential to prioritize positive influencing factors and address patient-specific conditions related to adverse factors^8^. While these factors are relevant to medication adherence in general, exploring their specific influences on hypertensive treatments could help to identify and resolve adherence-related issues, which would also help patient engagement in managing the condition^1^. At this point, there is a need to focus on innovative concepts, such as patient involvement/shared decision-making and timing of medication taking^10,11^, which have been identified as particularly relevant to hypertension management due to the complexity of treatment regimens and the chronic nature of the disease^2^.

Patient involvement, a term often used interchangeably with patient participation, pertains to collaborative decision-making between patients and healthcare providers regarding care and treatment options^12^. After the paradigmatic change in healthcare – which ascribes to patients an active rather than passive role^13^, it has become increasingly important to involve them in the decision-making process and consider their preferences^14^. This fundamental shift provides the motivation for emphasizing patient involvement, greater patient participation in medication decisions, and optimal medication regimen timing to enhance hypertension adherence. Recent studies have suggested that medication timing should be based on the optimum adherence time, and it may be beneficial to involve patients in this planning^15–17^. For instance, the study conducted by Mackenzie et al. (2022) investigated treatment adherence in two groups of patients, one with antihypertensive medication intake in the morning (06:00–10:00) and the other in the evening (20:00–00:00). The study found that the evening group had significantly lower adherence levels than the morning group^16^. In contrast, the HYGIA study demonstrated that the time of day had no effect on adherence^15^. Although studies were conducted on the time of taking antihypertensive drugs, the results were inconsistent^15–17^. Thus, there is a need for further research to determine whether patient involvement in identifying optimal medication times can in fact enhance adherence, thus resolving the discrepancies currently found in the literature^7^. Furthermore, little research has been done on the ways in which patient involvement affects decision-making for hypertension^10,18^. Resolving these issues in the literature is important since hypertension treatments often necessitate long-term medication adherence, and more active patient involvement could enhance consistency in following treatment regimens^11^. The existing literature examines the impact of medication timing separately from patient involvement on treatment adherence. However, the simultaneous examination of these variables can offer valuable insights into the complex nature of treatment adherence, which is shaped by individual preferences and environmental influences^7,9–11^. Research demonstrates that shared decision-making and participation in medication-related choices have the potential to enhance adherence^10,18^. Patients are more inclined to adhere to a treatment regimen when they play a role in its design and when it aligns with their lifestyle. Furthermore, involving patients in these decisions allows them to tailor their treatment schedules to their daily routines, resulting in more consistent adherence. This integrated approach also addresses the psychological dimensions; medication schedules corresponding to patients’ lifestyles are less likely to be forgotten or overlooked^7–10,18^. We can better understand adherence by investigating medication timing and patient involvement, two crucial factors in developing personalized and effective treatment strategies. Therefore, the explicit focus of this study is to examine how these two factors combined influence hypertensive treatment adherence, which is a critical aspect of managing this chronic disease.

### Aim and objectives

This study aimed to determine the potential contributions of medication timing and patient involvement to hypertension patients’ treatment adherence. Additionally, it examined patients’ preferences regarding their involvement in treatment decisions and factors encouraging active participation in shared decision-making.

## Materials and methods

### Study design

A descriptive cross-sectional study was conducted between May and August 2023, following the STROBE (Strengthening the Reporting of Observational Studies in Epidemiology) Statement.

### Setting

The study was carried out at a major university hospital located in the city of Izmir, Turkey. The hospital’s structure is similar to other university hospitals in Turkey. This hospital has a large capacity and serves a significant portion of the population, thus the choice of institution was critical in ensuring the findings were relevant to a wide range of patients.

### Sampling and participants

The sample size was calculated using the G Power 3.0.10 program (Institut für Experimentelle Psychologie, Heinrich Heine Universität, Düsseldorf, Germany) based on Irshaidat et al.‘s study (2023) data^19^. To ensure a larger sample and thus sufficient data, resulting in meaningful relationships, the effect size of 0.20 was chosen with a power of 0.95, alpha of 0.05, and a sample size of 272^20^. Assuming a 10% non-response rate, the research team aimed to recruit 299 patients. The study included patients diagnosed with hypertension based on office blood pressure (blood pressure taken in a clinical setting using standard protocol values) BP ≥ 140/90 (according to the 2024 European Society of Cardiology Guidelines^1^), all using the same antihypertensive medication for at least a month (ensuring consistency and enabling a more precise evaluation), taking medications between 06.00 and 10.00 in the morning and 20.00- midnight, and aged 18 years and over. The following were excluded from the sample: those with hearing problems, those using split doses, and those diagnosed with stroke and dementia. Of the 451 individuals screened for eligibility to participate in the study, 60 were excluded for using split doses, 30 for using drugs for less than one month, and 57 for cognitive and auditory deficiencies. The final sample size was 304 patients.

### Data collection tools

*Patient Information Form *was created to gather information on patient socio-demographics and clinical characteristics, including age, gender, educational status, co-living, medical history, previous cardiovascular events, duration of hypertension, number of hypertension medications, medication-taking time, and patients’ preferred time for medication. Medication timing was operationalized as a variable by categorizing patients into two groups based on medication time. The morning group consisted of those who took their medications between 06:00 and 10:00, and the evening group consisted of those who took their medications between 20:00 and midnight. This classification follows previous research recommendations, which similarly operationalized medication timing to explore its impact on treatment adherence^16^.

We additionally gathered information about patients’ involvement preferences in decision-making and the factors that encourage them to participate more actively. Patient involvement was assessed using the *Control Preferences Scale (CPS)*, originally developed and validated by Degner and later modified by Bruera^21,22^. Participants’ preferences for involvement in decisions were made using the SCS, which offers five options: (1) Patients make decisions alone; (2) Patients make decisions after consulting providers; (3) Joint patient-provider decisions; (4) Providers make decisions after consulting patients; (5) Providers make treatment decisions alone^21,22^. We then grouped the responses into categories as active (answers 1 and 2), shared (answer 3), and passive decision-making (answers 4 and 5). Patients were also asked to indicate their level of agreement with each of the six factors encouraging/promoting involvement^23^. CPS and the list involving promoting factors were translated into Turkish following the International Society for Pharmacoeconomics and Outcome Research (ISPOR) guidelines^24^.

The *Medication Adherence Report Scale (MARS)*, created by Horne and Hankins, evaluates medication adherence via a 5-point scale ranging from 5 (never) to 1 (very often)^25^. The MARS scale assesses patients’ self-reported responses to items related to issues about medication adherence highlighted in the background section. These included patients missing doses, forgetting doses, altering the prescribed dosage, stopping medication without consulting a healthcare provider, and taking smaller doses than instructed. Possible overall scores range between 5 and 25, with higher scores indicating better adherence. The outcome variable was dichotomized in logistic regression analyses, according to the total score on MARS-5, where adherence was defined as scores 23–25 and non-adherence as scores 5–22^19^. Sen et al. validated its Turkish version in 2019, achieving a Cronbach alpha of 0.78^26^. The present study’s Cronbach alpha coefficient was 0.76.

### Data collection procedure

The convenience sampling method was used to select participants from inpatients in the hospital cardiology unit. Eligible patients were identified based on their medical records and approached by the research team in person. After being informed about the study verbally, they were asked to approve the informed consent form. Participants were then provided with a survey packet to complete independently. If necessary, they could request authors to read aloud and clarify questions. The survey was completed on paper, and self-reports were checked against medical records to validate the medication information. The data collection process was conducted by the authors, and the survey took approximately 10–15 min to complete.

### Data analysis

We utilized the Statistical Package for the Social Sciences (SPSS) v. 24.0 by IBM Corp to analyze the patient data in detail. Descriptive statistics were employed to outline the patients’ socio-demographic and medical characteristics. We conducted binary logistic regression analyses to explore the relationships between treatment adherence and covariates. The selection of covariates to include in the regression analysis was based on univariate analyses via chi-square, and it was decided to include variables with a significance level of *p* <.25 This level was chosen because the traditional *p*<.05 threshold risks excluding potentially essential variables, whereas this more flexible threshold helps reduce the likelihood of overlooking these^27^. Additionally, we followed recommendations for appropriate variable selection in logistic regression to ensure rigor and avoid pitfalls^28^. Accordingly, in addition to the two main variables, i.e., patient preferences and medication timing, the following were included in the model, with *p* <.25: age, educational status, previous cardiovascular events, duration of HT, and number of HT medications. We used the ‘enter’ method to select the variables included in the model. The regression model used standardized β and 95% confidence interval (CI) to determine the strength of the independent variables. The significance level was set at *p* <.05. Hosmer–Lemeshow Goodness of Fit tests were used to select the best prediction model.

### Ethical considerations

Ethical approval was obtained from the Non-Interventional Research Ethics Committee (Decision Date: April 12, 2023, Decision Number: 2023/12 − 01) and institutional permission from the hospital where the study was conducted. The patients participating in the study were informed about the study and provided both written and verbal consent. The investigation conforms with the principles outlined in the Declaration of Helsinki.

## Results

### Participants’ demographic and medical characteristics

Of the participants, over half were male (53.9%), and 64.8% were over 65. Nearly half (44.4%) had completed education to high school or above. The vast majority lived with their families (88.2%). The most common conditions were arrhythmia (44.7%) and diabetes mellitus (40.8%). Nearly half the patients (44.1%) had experienced a previous cardiovascular event. Most had first been diagnosed with hypertension more than five years previously (77.3%). Slightly over half of the patients, 50.3%, were taking more than two medications for hypertension. Furthermore, 61.8% of patients’ medication time was between 6 and 10 a.m. However, as many as 90.8% of participants stated a preference for morning medication (Table 1).

**Table 1:** Characteristics of patients with hypertension (*n* = 304).

Demographic and clinical characteristics	*n*(%)
Age (years)	
< 65 years	107(35.2%)
≥ 65 years	197(64.8%)
Gender	
Female	140(46.1%)
Male	164(53.9%)
Education	
Elementary or middle school	169(55.6%)
High school or above	135(44.4%)
Co-living	
Alone	36(11.8%)
Family	268(88.2%)
Medical history∗	
Diabetes mellitus	124(40.8%)
Kidney failure	23(7.6%)
Sleep apnea syndrome	11(3.6%)
Heart failure	95(31.3%)
Arrhythmia	136(44.7%)
Others∗∗	26(8.6%)
Previous cardiovascular events (Yes)***	134(44.1%)
Duration of hypertension	
1–5 years	69(22.7%)
≥ 5 years	235(77.3%)
Number of hypertension medications	
1	151(49.7%)
≥ 2	153(50.3%)
Medication time****	
All drugs in the morning	188(61.8%)
≥ one drug in the evening	116(38.2%)
Patients’ preferred medication time	
In the morning	276(90.8%)
In the evening	28(9.2%)

^∗^n can take multiple values.

^∗∗^Others: Hyperlipidemia, systemic lupus erythematosus, hypothyroidism.

***Previous cardiovascular events: myocardial infarction, stable or unstable angina.

****In the morning (06.00–10.00), In the evening (20.00–00.00).

### Participants’ involvement preferences and encouraging factors

Of all the patients, 82 (27.0%) preferred a shared decision-making approach, while 183 (60.2%) preferred a passive approach to their treatment. Only 39 patients (12.8%) wanted to make treatment decisions independently. All patients were able to express their preferences and were willing to share their preferences for participating in the treatment decision-making process. Regarding the factors that would enhance participation in decision-making, a clear majority (80.9%) cited the opportunity to determine medication times that fit their existing daily routines (e.g., before breakfast or after coffee). Additionally, patients agreed that they would participate if they received encouragement from health professionals (69.4%), knew about treatment options (53.6%), or had adequate consultation time with health professionals (67.8%). However, only 12.2% of patients preferred more innovative and engaging methods while receiving health information, and 42.4% believed that simpler language should be used to share information (Table 2).

**Table 2:** Characteristics of involvement of patients with hypertension (*n* = 304).

Patient involvement preferences (Control Prefences Scale - CPS) *n(%)*			
Active involvement	39(12.8%)		
Shared decision-making	82(27.0%)		
Passive preference	183(60.2%)		
**Factors that would encourage participation**	Agree	Disagree	Not sure
Planning by the daily routine	246(80.9%)	24(7.9%)	34(11.2%)
Information about treatment	163(53.6%)	58(19.1%)	83(27.3%)
Positive attitudes of healthcare professionals	211(69.4%)	58(19.1%)	35(11.5%)
The health information is delivered in an engaging way, such as through pamphlets or videos	37(12.2%)	101(33.2%)	166(54.6%)
Allocation of adequate consultation time with the provider	206(67.8%)	84(27.6%)	14(4.6%)
Information is far from medical language and is understandable	129(42.4%)	73(24.0%)	102(33.6%)

### Univariate analyses

Results from the univariate analysis revealed that the following were significantly associated with treatment adherence: education, previous cardiovascular events, duration of hypertension, and preferences for involvement in decision-making. Participants with a higher level of education exhibited higher scores for the MARS items (72.6% vs. 55.0%; *p* =.002), as did those diagnosed with hypertension for more than five years (65.1% vs. 45.7%; *p* =.026) and patients with no prior cardiovascular events (69.4% vs. 54.5%; *p* =.007). Additionally, significantly higher MARS item scores were found for patients actively involved in shared decision-making than those who were passive (81.0% vs. 50.8%; *p* =.000). No significant associations were found between treatment adherence scores and age, gender, co-living status, number of hypertension medications, and medication timing (Table 3).

**Table 3:** Association between adherence scores and demographic characteristics, patient preferences, and medication timing (*n* = 304).

	Adherence	Non-adherence		Fisher or χ2	*p*
Age group					
< 65 years	60(56.1%)	47(43.9%)		3.225	0.073
≥ 65 years	131(66.5%)	66(33.5%)			
Gender					
Female	88(62.9%)	52(37.1%)		0.000	0.993
Male	103(62.8%)	61(37.2%)			
Education					
Elementary or middle school	93(55.0%)	76(45.0%)		9.912	**0.002**
High school or above	98(72.6%)	37(27.4%)			
Co-living					
Alone	21(60.0%)	14(40.0%)		0.136	0.713
Family	170(63.2%)	99(36.8%)			
Previous cardiovascular events					
Yes	73(54.5%)	61(45.5%)		7.156	**0.007**
No	118(69.4%)	52(30.6%)			
Duration of hypertension					
1–5 years	16(45.7%)	19(54.3%)		4.961	**0.026**
≥ 5 years	175(65.1%)	94(34.9%)			
Number of hypertension medications					
1	100(66.2)	51(33.8)		1.482	0.223
≥ 2	91(59.5)	62(40.5)			
Medication timing					
All drugs in the morning	117(62.2%)	71(37.8%)		0.075	0.785
≥ 1 drug in the evening	74(63.8%)	42(36.2%)			
Patient involvement preferences (CPS)					
Active and shared involvement	98(81.0%)	23(19.0%)		28.393	**0.000**
Passive performance	93(50.8%)	90(49.2%)			

χ2 Chi-square.

P<0.05; bold values are statistically significant.

### Multivariate analyses

Table 4 shows the results of binary logistic regression analysis for factors predicting medication adherence. Patients with no previous cardiovascular events were more likely to be adherent to the antihypertensive therapy than the others (OR = 2.465; [95% CI = 1.444–4.208; *p* =.001]). Those with hypertension duration ≥ 5 years showed more adherence than those with hypertension duration < 5 years (OR = 3.953; [95% CI = 1.624–9.623; *p* =.002]). Patients who had active and shared preferences on involvement in decision-making were more likely to be adherent to passive preferences (OR = 5.649; [95% CI = 2.528–12.622, *p* =.000]). However, treatment adherence scores was not found to be related with age, educational status, the number of hypertension medications used, and medication timing.

**Table 4:** Results of the multivariate binary logistic regression analysis on the relationship between adherence scores and influencing factors (*n* = 304).

Influencing factors	β	SE	OR (95% CI)		*p*
Age (Reference: <65 years)	0.358	0.287	1.430(0.815–2.509)		0.212
Education (Reference: Elementary or middle school)	0.105	0.385	1.110(0.522–2.361)		0.786
Previous cardiovascular events (Reference: Yes)	0.902	0.273	2.465(1.444–4.208)		**0.001**
Duration of hypertension (Reference: 1–5 years)	1.375	0.454	3.953(1.624–9.623)		**0.002**
Number of hypertension medications (Reference: ≥2)	0.313	0.291	1.367(0.772–2.419)		0.283
Medication timing (Reference: ≥1 drug in the evening)	0.051	0.295	1.052(0.591–1.875)		0.863
Patient involvement preferences (CPS) (Reference: Passive)	1.731	0.410	5.649(2.528–12.622)		**0.000**

β, regression coefficient; CI, confidence interval; OR, odds ratio; SE, standard error.

Reference refers to the baseline category against which other categories are compared in the analysis.

*p* <.05; bold values are statistically significant.

## Discussion

This study explored medication adherence factors, focusing on medication timing and patient involvement in decision-making. Our findings indicate that patient involvement was the key factor; patients reported greater engagement when their medication regimen aligned with their daily routines, and when healthcare providers fostered a welcoming environment. While medication timing did not directly predict adherence responses, most participants expressed a preference for the morning. Adherence was additionally associated with complementary factors such as prior cardiovascular events and the duration of hypertension. These findings open new avenues for understanding medication adherence, highlighting the role played by the treatment plan and the patient’s level of involvement in their care.

Healthcare providers should consider the potential impact on patient adherence when changing medication regulations. Non-adherence to medication can lead to the progression of chronic diseases^29^, and it is therefore essential for healthcare providers and managers to evaluate the relevant factors. Most participants in this study were older adults, therefore healthcare providers should consider the needs of this age group and tailor interventions accordingly, taking into account factors which participants in this study indicated as important. These include adequate consultation time, using clear language for sharing information, and aligning medication times with daily routines. Studies support these approaches, with research showing that older adults benefit from clear, simple communication and medication schedules aligned with their daily routines to improve adherence^30,31^.

Our research has shown that those with experience of previous cardiovascular events are less likely to adhere to their treatment regimen. This is consistent with Wan et al.’s (2022) study^30^, which found generally better adherence for patients with fewer complications. The duration of hypertension may also influence medication adherence in various ways. The recent study found that patients with five or more years of hypertension had significantly higher MARS scores, which aligns with previous studies showing a positive correlation between hypertension duration and adherence^32,33^. In contrast, Chu et al. (2021) suggested that patients with hypertension over 9.5 years were more likely to have lower adherence^34^. These contradictory findings indicate that the relationship between duration and adherence may be complicated by factors such as patients’ disease knowledge, awareness of adherence’s positive impact on blood pressure control and complication prevention, and psychological barriers, such as adherence fatigue^31^.

In addition to the longevity of hypertension, another issue that has gained global attention is the role of medication time preferences. Although this issue made no significant difference in the current study, prior research on antihypertensive medication has indicated better adherence by those taking morning medication compared to those taking evening medication. In this study, 61.8% of patients reported taking their medication between 6 and 10 a.m.; however, the overwhelming majority, 90.8%, expressed a preference for mornings. These findings, therefore, highlight the relevance of medication timing; however, timing may not be the sole determinant, as the study found no significant relationship between medication timing and adherence. This anomaly may be attributed to several factors. In addition to timing, patient adherence is influenced by a wide range of contextual factors, such as individual behaviors and psychological factors, and practical aspects, such as the complexity of the medication regimen^7^. In this study, adherence patterns were strongly influenced by prior cardiovascular events, the duration of hypertension, and the level of involvement in treatment decision-making. Thus, while medication timing has potential influence, adherence is likely to be additionally shaped by a range of patient-specific factors, healthcare-related variables, and personal preferences^17^. Therefore, while relevant, medication timing should not be viewed in isolation as the primary intervention for improving adherence; rather, in this regard, it is important to consider the interaction between medication timing and other key factors, such as treatment routines and patient involvement in decision-making.

In recent years, there has been a shift towards a more collaborative approach to clinical decision-making, with the patient playing a more central role in partnership with the medical staff^13^. However, our study found that many patients still prefer a passive role in decision-making, possibly due to the longstanding paternalistic approach in healthcare organizations. Even healthcare providers who in general aim to promote shared decision-making may sometimes resort to a paternalistic approach when discussing treatment options^35^. Logistic regression analysis showed that patients who participated in shared and active decision-making had significantly higher adherence responses, highlighting the importance of ongoing initiatives to increase patient involvement. The findings from our study suggest that the patient involvement-adherence relationship is mediated by factors such as the alignment of medication timing with daily routines and healthcare provider encouragement. These two factors are critical in enhancing patient engagement, thereby improving adherence^2,10^. Interestingly, these findings may overlap with results for other long-term health conditions, with similar implications for decision-making involvement and greater treatment adherence^36,37^. To overcome the continued dominance of the paternalistic approach, healthcare professionals should prioritize communication, education, and viewing healthcare as a partnership^38^. Additionally, these processes can be strengthened by improving health literacy, providing access to reliable information, and offering training for decision-making involvement^39^.

Our findings on patient involvement align with aspects that can promote shared decision-making^39^. One significant factor motivating participation is the alignment of decisions with patients’ daily routines because medication timings are often associated with these routines, such as mealtimes, in treatment management^40^. Previous research suggests that when treatment aligns with a patient’s established daily schedule, medication adherence can improve, as patients are more likely to follow regimens that fit naturally into their routines^7^. Healthcare providers can consider an adaptive approach by suggesting medication timings that suit patients’ daily routines, such as before breakfast or after morning coffee. Healthcare professionals’ positive attitudes and adequate time allocation can also encourage patient participation in decisions^41,42^. Effective communication skills and a welcoming attitude are crucial in fostering the patient-provider relationship, which is pivotal in implementing shared decision-making^23^. However, clinicians need to recognize that not all patients are prepared for shared decision-making and may require support to increase their willingness to take responsibility – highlighting the importance of advocating for patient involvement through personalized strategies that align with their preferences. Thus, patient involvement should be regarded as a general principle, and at the same time, an adaptable process that requires personalization. This approach can mitigate passive decision-making, often associated with a paternalistic approach^41^.

### Limitations

It is important to note that the data collected in this study reflects participants’ perceptions, therefore any findings should be interpreted as associations between the explored variables rather than causality. Future longitudinal studies could help establish clearer causative links. The patients involved in the study were selected from a university hospital, so the results may not apply to other healthcare settings, such as primary care centers, and these other healthcare organizations could be a focus in future studies. Regardless of this limitation, the research reached the required number of patients recommended in power analyses, which strengthens the accuracy of the results. Furthermore, it is worth noting that, in our country, most university hospitals have a similar structure, which adds to the generalizability of the findings.

### Relevance for clinical practice

It is crucial to ensure that patients take their medication as prescribed to achieve better healthcare outcomes. Our research found that involving patients in the decision-making process is a key factor in tailoring medication regimens to individual needs and promoting adherence. Since collaboration among healthcare providers is necessary for patient involvement, healthcare managers should consider offering incentives to increase awareness among both providers and patients. However, medical staff should be aware that not all patients are ready for active participation in decision-making, and should provide support when necessary^41,43^. Patients preferring a more passive and paternalistic approach, particularly older adults, may struggle to engage fully with their treatment plan, and this may limit their adherence. In mitigation, healthcare providers could offer support by facilitating a more gradual approach to engagement by providing clear explanations of treatment options, addressing concerns, and actively attending to patients’ preferences. Encouraging dialogue and providing information about treatments would be beneficial, helping patients to feel more comfortable taking an active role in their care. By adapting treatment options to align with patients’ daily routines, such as integrating medication schedules with mealtimes or other daily events, healthcare providers can improve adherence and foster a stronger patient-provider relationship.

There is currently no clear consensus among medical guidelines on the optimal timing for administering different antihypertensive drugs, leading to varying clinical practices. More research is required to establish a consensus on drug timing. In the meantime, it is recommended that anti-hypertensive medication is scheduled for times when patients are most likely to take them regularly and experience the least side effects. Although patients generally prefer the morning, research shows that adherence is not significantly affected by timing. However, establishing a morning routine is generally easier^17^. Therefore, prescribing morning medications may be beneficial in minimizing patients’ medical issues or adherence challenges.

## Conclusions

The involvement of patients was found to be a significant factor in adhering to medication, unlike the timing of medication administration. When patients were more involved in decision-making, there was a higher likelihood of adhering to medication. Two key factors in encouraging patient involvement were identified: compliance with daily routines and healthcare professionals’ positive attitudes.

## Data Availability

The datasets used and analyzed during the current study are available from the corresponding author on reasonable request.
